# Altered Brain Function and Network Topology in Patients With Acromegaly: Resting‐State fMRI Study of Networks Related to Cognitive and Emotional Processing

**DOI:** 10.1002/cns.70755

**Published:** 2026-01-19

**Authors:** Zerui Wu, Xuejie Yu, Yingyue Zhang, Jinming Yang, Qilin Zhang, Shun Yao, Xuefei Shou, Xiang Zhou, Yongfei Wang, Hao Li, Liguo Jia, Yifei Yu, Weiwei Wang, Zengyi Ma, Wenqiang He

**Affiliations:** ^1^ Department of Neurosurgery First Affiliated Hospital of Wenzhou Medical University Wenzhou China; ^2^ Department of Neurosurgery Huashan Hospital, Shanghai Medical College, Fudan University Shanghai China; ^3^ Neurosurgical Institute of Fudan University Shanghai China; ^4^ Shanghai Key Laboratory of Brain Function Restoration and Neural Regeneration Shanghai China; ^5^ National Center for Neurological Disorders Shanghai China; ^6^ Department of Nursing, Huashan Hospital, Shanghai Medical College Fudan University Shanghai China; ^7^ Department of Radiology Huashan Hospital, Shanghai Medical College, Fudan University Shanghai China; ^8^ Human Phenome Institute Fudan University Shanghai China; ^9^ Department of Endocrinology, Huashan Hospital, Shanghai Medical College Fudan University Shanghai China

**Keywords:** acromegaly, cognitive impairment, functional connection, graph theory, multiple kernel learning, resting‐state functional magnetic resonance imaging

## Abstract

**Context:**

Neurodegenerative diseases are particularly prevalent among patients with acromegaly, but their functional alterations remain poorly understood.

**Objective:**

To explore the neurobiological mechanisms of excess growth hormone (GH) on brain functional activity and connectivity in acromegaly.

**Methods:**

Neuropsychological assessments and resting‐state functional magnetic resonance imaging (fMRI) were conducted on 27 patients with acromegaly and 25 healthy controls. The amplitude of low‐frequency fluctuations (ALFF), fractional ALFF (fALFF), and regional homogeneity (ReHo) were compared between groups via voxel‐based analyses, while graph theory was used to assess brain network topology. *T*‐tests and multikernel support vector machine (MK‐SVM) were used to identify discriminative connectome features for classification.

**Results:**

Patients with acromegaly exhibited lower Montreal Cognitive Assessment scores, increased ALFF in the default mode network regions, and decreased fALFF in the frontal–parietal control network areas. ReHo was elevated in the visual network but reduced in the frontal–parietal network. Disruptions were observed in key hub nodes within the default mode and visual networks. The MK‐SVM achieved 85.11% accuracy and 80.00% sensitivity in classifying patients.

**Conclusions:**

Patients with acromegaly exhibited altered brain function and network disruptions. These results offer novel insights into the mechanisms of excess GH in the brain.

## Graphical abstract

Patients with acromegaly show disrupted brain function and network connectivity, particularly in the default mode and frontal–parietal networks. This study reveals how excess GH and IGF‐1 alter brain activity, offering new insights into the neurobiological mechanisms underlying cognitive decline in acromegaly.

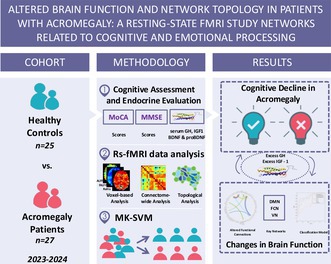

## 1 Introduction

Acromegaly is a rare endocrine disorder characterized by chronic growth hormone (GH) hypersecretion, typically due to pituitary adenomas [1]. This disease often progresses insidiously, with an average diagnostic delay of 4–10 years or longer [2]. Prolonged elevations of GH and its target hormone, insulin‐like growth factor 1 (IGF‐1), can adversely affect various organ systems, resulting in bone overgrowth, cardiovascular complications, and metabolic dysfunction. Emerging evidence additionally suggests that excess GH/IGF‐1 may also affect the central nervous system [3]. Such patients demonstrate an increased prevalence of cognitive deficits, memory impairment, and executive dysfunction, even after achieving biochemical remission [4]. However, the mechanisms behind these functional brain alterations in the setting of GH excess are poorly understood.

GH and IGF‐1 receptors are widely expressed in brain regions involved in neurogenesis and synaptic maintenance, particularly the hippocampus and prefrontal cortex [5]. Physiological levels of GH and IGF‐1 support brain maturation, neurogenesis, and neural protection [5], but chronic GH excess can cause pathological hyperphosphorylation of the hippocampal tau protein and subsequent cognitive impairment [6]. Recent studies have highlighted the role of brain‐derived neurotrophic factor (BDNF) and its precursor, proBDNF, in regulating synaptic plasticity, cognitive function, and neuronal survival [7]. An imbalance in the conversion of proBDNF to BDNF can significantly affect cognitive function and accelerate the progression of neurodegenerative diseases [8], but its relevance in the setting of chronic GH/IGF‐1 excess and cognitive impairment in acromegaly remains largely unexplored. Neuroimaging studies in patients with acromegaly have revealed microstructure alterations in both the gray and white matter [9], although these structural findings alone provide limited insight into the dynamic reorganization of functional brain networks.

Resting‐state functional magnetic resonance imaging (rs‐MRI) is a powerful tool for mapping intrinsic brain connectivity through blood‐oxygen‐level‐dependent (BOLD) signal fluctuations at rest [10]. This approach has revealed aberrant functional networks across various endocrine disorders such as Cushing's disease [11]. Moreover, recent advancements in computational neuroscience, particularly graph theory and machine learning algorithms such as Multiple Kernel Support Vector Machine (MK‐SVM), have made it possible to quantify global and nodal network properties and identify disease‐specific biomarkers [12]. Although this new technology has revolutionized the study of neurodegenerative disorders [13, 14], their application in understanding neural network alterations in acromegaly remains largely unexplored.

This study compared the cognitive performance and serum BDNF and proBDNF levels between treatment‐naïve patients with acromegaly and healthy controls. Alterations in brain functional activity and topological network changes were investigated via rs‐fMRI combined with advanced computational approaches. An MK‐SVM classification model was developed to delineate the core affected neural features. This work aims to provide novel insights into how chronic GH/IGF‐1 excess remodels the brain network architecture, potentially uncovering biomarkers for the early detection of CNS involvement in acromegaly.

## 2 Methods

### 2.1 Participants

This study enrolled 27 treatment‐naïve acromegaly patients admitted at the Department of Neurosurgery at Huashan Hospital between 2023 and 2024. The inclusion criteria were as follows: (1) confirmed diagnosis of acromegaly based on the 14th Acromegaly Consensus Conference (i.e., serum IGF‐1 > 1.3 times the upper limit of [ULN] for age, and failure to suppress serum GH levels below 1 mg/L after a 75‐g oral glucose tolerance test) [15]; (2) age 18–60 years old; (3) MRI evidence of pituitary adenoma. The healthy control group included 25 sex‐ and age‐matched healthy individuals. All participants were right‐handed Chinese Han individuals. The exclusion criteria for both groups were as follows: (1) history of stroke, traumatic brain injury, or other intracranial lesions; (2) neuropsychiatric disorders, including Alzheimer's disease, Parkinson's disease, anxiety, and depression; (3) severe systemic disease, such as heart failure, renal failure, and liver disease; (4) sensory impairments (visual or auditory) precluding completion of the neuropsychological assessment; (5) MRI contraindications, including claustrophobia and pacemaker implantation.

### 2.2 Neuropsychological Assessment

Cognitive function was evaluated by experienced neurologists using the Mini‐Mental State Examination (MMSE) and the Montreal Cognitive Assessment (MoCA). MoCA scores below 26 indicated potential cognitive impairment.

### 2.3 Blood Sample Collection and Measurement

Venous blood samples were drawn between 06:00 and 10:00 a.m. after 10–12 h of fasting. Serum samples were isolated via centrifugation (3000 rpm for 10 min at 4°C) and stored at −80°C for subsequent enzyme‐linked immunosorbent assay (ELISA) assessment. Serum GH and IGF‐1 levels in patients with acromegaly were analyzed using the IMMULITE 2000 immunoassay system (Siemens, Germany). Serum concentrations of BDNF (Cat No. ml026032) and proBDNF (Cat No. ml029799) were measured using an ELISA kit (mlbio company, Shanghai, China) according to the manufacturer's instructions.

### 2.4 Rs‐fMRI Data Acquisition and Preprocessing

MRI was performed using a clinical 3‐T scanner (Siemens Healthcare, Erlangen, Germany) with a 64‐element head coil at Huashan Hospital. A high‐resolution T1‐weighted 3D magnetization prepared rapid acquisition gradient echo sequence covering the whole brain was applied for anatomic reference with the following parameters: repetition time (TR) = 8.156 ms, echo time (TE) = 3.18 ms, flip angle (FA) = 9°, matrix size = 256 × 256, field of view (FOV) = 250 × 250 mm, slice thickness = 1.0 mm, gap = 0 mm. The rs‐fMRI data were obtained for 8 min using a gradient‐recalled echo‐planar imaging (GRE‐EPI) sequence with the following parameters: TR = 2000 ms; TE = 30 ms; FA = 90°; acquisition matrix = 64 × 64; FOV = 220 × 220 mm; thickness = 3.2 mm; gap = 0 mm. During the scan, all participants were instructed to keep their eyes closed but remain awake, while maintaining a relaxed state and minimizing movement for optimal data acquisition. None of the participants reported falling asleep during the scanning process.

The MRI data was preprocessed via statistical parametric mapping (SPM12, http://www.fil.ion.ucl.ac.uk/spm) [16] and the Data Processing Assistant for rs‐fMRI (DPABI, http://rfmri.org/dpabi) [17]. To reduce signal instability and the impact of the subjects' adaptation to the scan, the first 10 time point images were removed. Corrections for time and head movement were applied as well. All images were aligned to the middle layer to eliminate small differences between layers caused by different acquisition time points, while a six‐parameter rigid body transformation was used to correct for head movement. Subjects with head movement parameters exceeding 3 mm and 3° were excluded. For spatial standardization, the realigned MRI images were spatially normalized using the T1 structural images of each subject as a reference. Subsequently, all images were resampled to isotropic voxels with a 3‐mm resolution. Data linear drift was removed, and time‐domain bandpass filtering was performed (0.01–0.08 Hz). Lastly, interference signals, including six head movement parameters, white matter, cerebrospinal fluid, and whole brain mean signal, were removed.

The Automatic Anatomical Labeling (AAL) atlas was used to measure the amplitude of the low‐frequency fluctuations (ALFF), fractional ALFF (fALFF), and regional homogeneity (ReHo) [18]. The specific computational methods were as follows: For ALFF, the images were smoothed with a Gaussian kernel with a full‐width‐half‐maximum (FWHM) of 6 mm. The ALFF values were calculated by finding the mean of the square root of the power spectrum, derived via fast Fourier transform. To obtain fALFF, the ALFF value was divided by the power spectrum of the entire frequency domain. For ReHo, the Kendall consistency coefficient was calculated for each voxel and its adjacent voxels in the time series, then smoothed using a Gaussian kernel with a 6‐mm FWHM after calculating the metrics. To improve data normality, Fisher's Z‐transform was applied to obtain the Z‐distribution maps of ALFF, fALFF, and ReHo for each participant, for subsequent statistical analysis.

### 2.5 Functional Network Construction and Network Analysis

First, the subjects' brains were segmented into 90 brain regions using the AAL atlas, serving as 90 brain functional network nodes. To facilitate the functional interpretation of the network features, the Yeo 7‐network parcellation template was overlaid on the AAL atlas [19]. Each cortical AAL node was assigned to the specific Yeo network that exhibited the maximal spatial overlap with that node. Subcortical structures within the AAL atlas (e.g., Thalamus, Caudate) were classified into a separate Subcortical Network based on anatomical definitions [20, 21]. The Pearson correlation coefficients were calculated for the mean time series between each pair of brain regions. As a result, this process generated a comprehensive functional connectivity matrix for each participant, representing the brain's overall functional connections as a 90 × 90 Pearson correlation matrix [22]. The threshold measure was defined using sparsity (S), which is the ratio of the actual to the maximum possible number of edges in the network. Since there is no universally accepted standard for selecting a single threshold, a range of thresholds was used in this study (0.05 ≤ S ≤ 0.50, with intervals of 0.01) [23, 24].

Whole‐brain network analysis was performed using the GRETNA toolbox3. To characterize the functional brain connectome, both global and nodal graph metrics were calculated. The global metrics were as follows. Modularity (*Q*) is defined as the degree to which the network can be decomposed into relatively independent sub‐networks, reflecting the functional segregation of the network. Global efficiency (*E*_global_) is the average inverse shortest path length between all pairs of nodes, which represents the overall capacity of the network for parallel information transfer and integration. Local efficiency (*E*_local_) is the efficiency of information exchange within the immediate neighbors of a node, indicating the capacity of the network for local information transmission and functional differentiation. The clustering coefficient (*Cp*) is the degree of cliquishness in a network, reflecting its local information transmission capability and functional differentiation capacity. For a specific node in the network, *Cp* is defined as the ratio of the actual number of edges existing between its neighboring nodes to the maximum possible number of edges among those neighbors. The overall *Cp* of the network is the average of the clustering coefficients of all nodes. The standardized cluster coefficient (*γ*) is the ratio of the observed clustering coefficient to that of a comparable random network [25]. The standardized feature path length (*λ*) is the ratio of the observed path length to that of matched random networks. The small worldness index (*σ*) is defined using the equation *σ* = *γ*/*λ*, wherein values greater than 1 indicate small‐world organization; this combines high local clustering with short path lengths for efficient global processing. Lastly, characteristic path length (*Lp*) is the average shortest path length between all pairs of nodes in the network, indicating the efficiency of global communication.

The assessed nodal graph metrics are described as follows. Betweenness centrality reflects how often a node lies on the shortest path between other nodes, indicating its importance as a communication hub. Degree centrality (DC), which represents the number of direct connections (i.e., edges) of a node, serves as a basic measure of nodal connectivity and is used to quantify the centrality of nodes within a network. Nodal *Cp* quantifies the local connectivity density among the neighbors of a node. Nodal efficiency (*E*_nodal_) measures the inverse shortest path length between a given node and all other nodes, indicating its ability to communicate efficiently across the network. Nodal local efficiency evaluates the efficiency of information exchange in the immediate neighborhood of a given node, which reflects the robustness of local processing. Nodal shortest path is defined as the average shortest path from a given node to all others, thereby describing its centrality in the network. Additionally, the area under the curve (AUC) was calculated for each network metric to provide a comprehensive scalar summary of the topological properties that is independent of the specific threshold selection.

### 2.6 Statistical Analysis

#### 2.6.1 Demographic, Endocrine, and Neuropsychological Data

The Shapiro–Wilk test was used to assess the normality of the continuous variables. Normally distributed variables were presented as the mean ± standard deviation and compared using Student's *t*‐test. Non‐normally distributed variables were displayed as median (interquartile range) and analyzed using the Mann–Whitney *U*‐test. Categorical variables were compared using chi‐squared tests. Pearson correlation analysis was performed to investigate the relationships between serum markers and cognitive function. Statistical analyses were conducted using SPSS 25.0.

#### 2.6.2 Brain Functional Index

To identify brain regions with significant differences, the ALFF, fALFF, and ReHo maps were compared between the two groups via Student's *t*‐test using the DPABI Advanced Edition Statistical Analysis tool (http://rfmri.org/dpabi). Age, gender, education, and BMI were entered as covariates. The results were corrected using a permutation‐based correction approach (10,000 permutations, voxel‐level, *p* < 0.01; cluster‐level, *p* < 0.01; two‐tailed) [26, 27]. Finally, multiple comparisons were adjusted using the Gaussian random field (GRF) correction method (*p* < 0.001).

#### 2.6.3 Network Metrics

To investigate the differences in graph theory metrics between the groups, two‐sample Student's *t*‐tests were conducted. A false discovery rate correction was applied to adjust for multiple comparisons, with statistical significance set at *p* < 0.05. In the analysis of functional connections, the results of the two‐sample Student's *t*‐test (*p* < 0.05) were used to select connections for comparing discriminative brain network connectome characteristics between two groups.

#### 2.6.4 Feature Selection, Consensus Connections and Classification

A straightforward approach was employed to mitigate challenges in determining the contribution of kernel combination techniques or feature selection to the final classification accuracy. Specifically, a *t*‐test with a threshold of *p* < 0.05 was used to select the nodal graph metrics and connection weights.

The LIBSVM toolbox for MATLAB was employed for SVM classification. Due to the limited sample size, a nested leave‐one‐out cross‐validation (nested LOOCV) strategy was used to ensure unbiased performance and minimize overfitting. In each outer loop, one case was left out as the unseen test case, while the inner loop was used for feature selection (the hyper‐parameter C for MK‐SVM). All feature selection and parameter optimization were performed exclusively within the training data to avoid data leakage. All connection features selected during the training process were recorded while evaluating the classification performance. The features selected via *t*‐test in each validation may differ. Consensus connections refer to features that are selected consistently across all validations [28, 29, 30], serving as the most discriminative features of brain networks. Thus, these consensus connections can be used to explore the pathological mechanisms and potential biomarkers associated with acromegaly.

Classification performance was compared using the functional connections (C), global metrics (G), and nodal metrics (N) of the brain network, as well as their combinations (i.e., C + G, C + N, G + N, and C + G + N). Multi‐kernel learning with a kernel combination approach was used to integrate multiple feature information. Further detailed information regarding the MK‐SVM can be found in previous studies [13].

## 3 Results

### 3.1 Demographics and Clinical Characteristics

Both groups were comparable in terms of age, sex, education, and body mass index (BMI) (all *p* > 0.05). Compared to controls, the acromegaly group had significantly lower MoCA and MMSE scores (*p* < 0.001), reduced serum BDNF levels (*p* < 0.001), and elevated proBDNF levels (*p* = 0.007). The clinical and demographic features of the participants are shown in Table 1.

**TABLE 1:** Demographic and clinical characteristics of the participants.

	Acromegaly (*n* = 27)	Controls (*n* = 25)	*p*
Age (years)	41.1 ± 13.9	42.2 ± 11.1	0.765^a^
Education (years)	13.3 ± 3.1	14.7 ± 2.1	0.068^a^
Gender (male/female)	7/20	11/14	0.245^b^
BMI	25.3 ± 2.8	24.0 ± 3.1	0.126^a^
Disease course (years)	4.2 ± 2.7	—	NA
Fasting GH (ng/mL)	18.9 (8.2–44.63)	—	
GH nadir (ng/mL)	10.4 (5–35.9)	—	NA
IGF‐1 index	2.2 ± 0.7	—	NA
IGF‐1 (ng/mL)	627.1 ± 183.9	—	NA
MMSE	28 (26–30)	30 (30–30)	< 0.001^c^
MoCA	26 (24–28)	30 (28–30)	< 0.001^c^
< 26 points	12 (44.4%)	0 (0%)	< 0.001^b^
BDNF	12.19 ± 2.22	14.95 ± 2.92	< 0.001^a^
proBDNF	4.45 ± 11.03	3.39 ± 1.63	0.007^a^
Hypothyroidism	0	—	NA
Hypocortisolism	0	—	NA

*Note:* Data are presented as means ± SD.

Abbreviations: BDNF, brain‐derived neurotrophic factor; BMI, body mass index; GH, growth hormone; IGF‐1, insulin‐like growth factor 1; MMSE, Mini‐Mental State Examination; MoCA, Montreal Cognitive Assessment Scale; proBDNF, precursor BDNF.

^a^ Unpaired *t*‐test, two‐sided.

^b^ Chi squared test, two‐sided.

^c^ Mann–Whitney *U*‐test, two‐sided.

**FIGURE 1:**
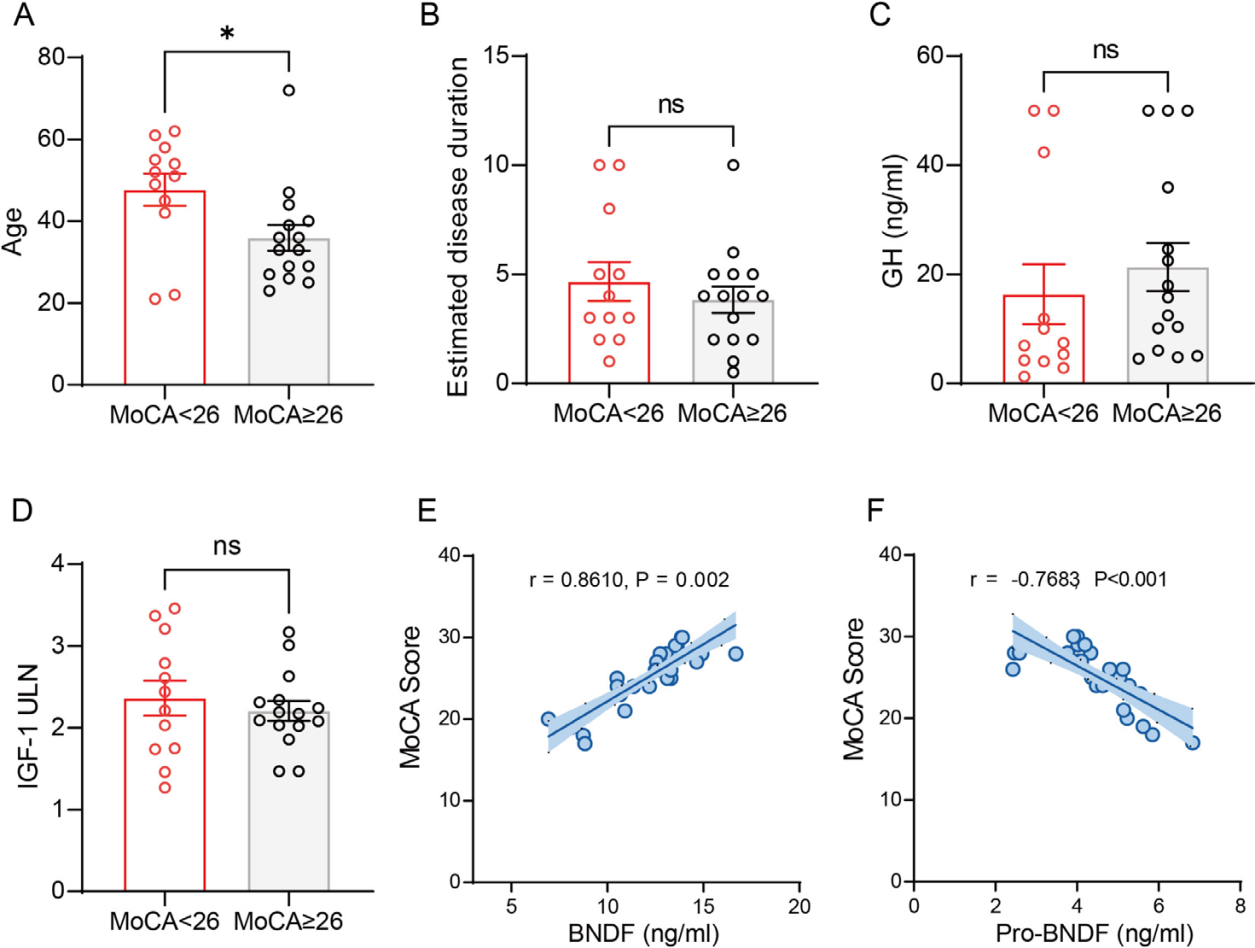
Analysis of clinical indices in patients with acromegaly. (A) Age distribution of acromegaly patients with MoCA < 26 and MoCA ≥ 26. *p < 0.05. (B–D) Disease duration (B), GH (C), and IGF‐1 index (D) between patients with MoCA < 26 and MoCA ≥ 26 (*p* > 0.05). (E, F) Pearson correlation shows a positive association between serum BDNF and MoCA scores and a negative association between serum ProBDNF and MoCA scores. GH, growth hormone; IGF‐1, insulin‐like growth factor‐1.

In the acromegaly group, 12 (44.4%) patients were screened positive for potential cognitive impairment (MoCA < 26). They were older than those with normal MoCA scores (Figure 1A, 47.7 ± 13.6 vs. 35.9 ± 12.2, *p* = 0.026). Two young female patients (21 and 22 years) also had MoCA < 26, both exhibiting high GH levels (50 and 46.11 ng/mL) and a relatively low IGF‐1 index (1.27× and 1.46× ULN). All patients demonstrated normal visual acuity and visual field examinations. Disease duration, GH and IGF‐1 index levels were comparable between the two subgroups (Figure 1B–F, *p* > 0.05). There was no significant correlation between serum GH (*r* = 0.073, *p* = 0.71) or IGF‐1 levels (r = 0.11, *p* = 0.586) and MoCA scores. However, serum BDNF levels were positively correlated with MoCA scores (*r* = 0.861, *p* = 0.002, Figure 1E), and proBDNF levels were negatively correlated with MoCA scores (*r* = −0.768, *p* < 0.001, Figure 1F).

### 3.2 ALFF, fALFF and ReHo in the Acromegaly and Control Groups

**FIGURE 2:**
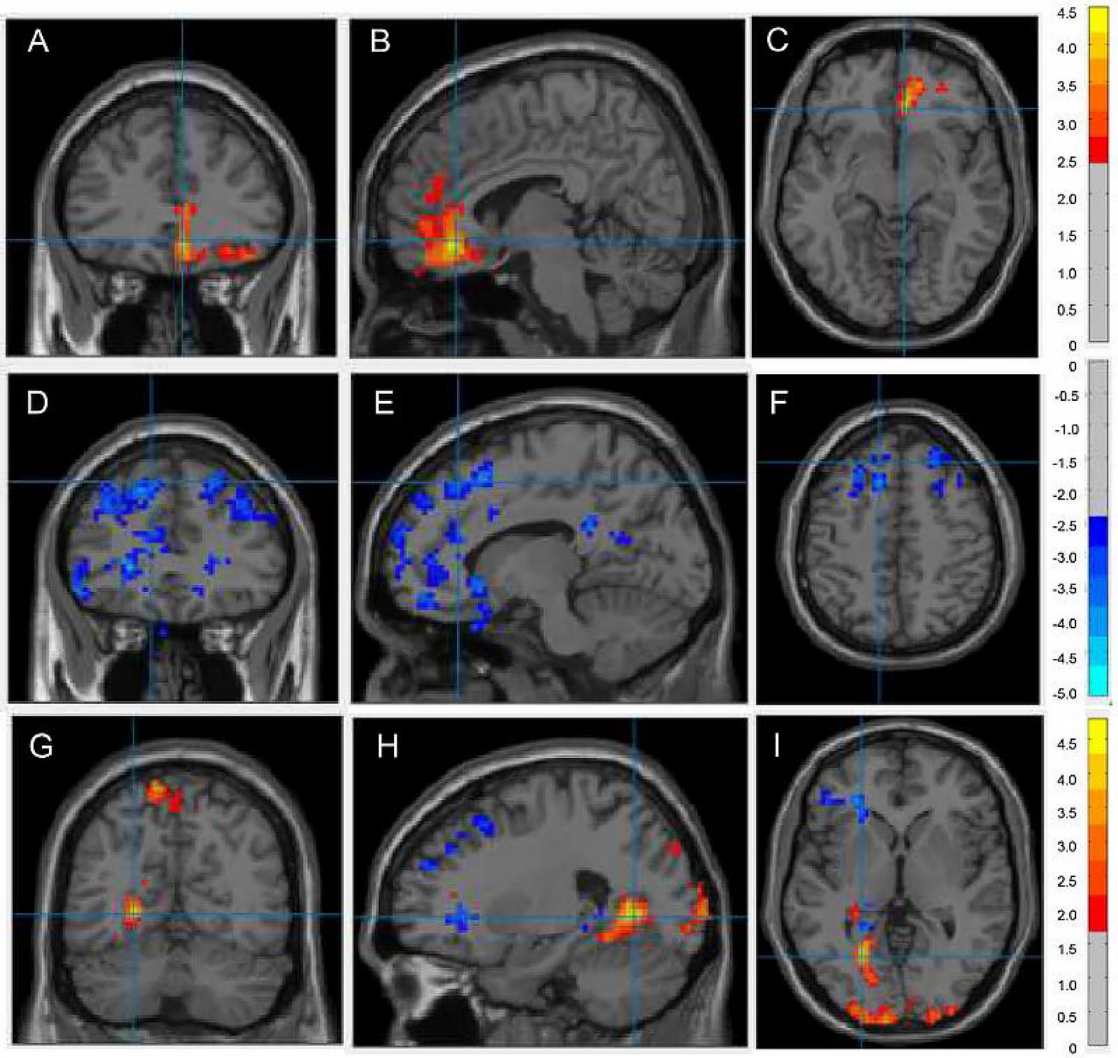
Acromegaly‐related alterations of ALFF, fALFF and ReHo. The red area highlights increased values (patients > healthy controls) and the blue area highlights decreased values (patients < healthy controls) in low frequency fluctuations (ALFF, A–C), fractional ALFF (fALFF, D–F), and regional homogeneity (ReHo, G–I).

Compared to controls, patients with acromegaly had increased ALFF values in the right prefrontal regions, including the medial orbital superior frontal gyrus (ORBsupmed), gyrus rectus (REC), anterior cingulate and paracingulate gyri (ACG), and orbital subdivisions of the superior/middle/inferior frontal gyri (ORBsup/ORBmid/ORBinf) (Table S1 and Figure 2A–C). Meanwhile, fALFF was reduced in the bilateral superior frontal gyrus, dorsolateral (SFGdor), middle frontal gyrus (MFG), and right precuneus (PCUN) among those with acromegaly (Table S1 and Figure 2D–F). Correlation analysis showed no significant relationship between serum GH, IGF‐1, BDNF, or ProBDNF levels and mean frontal ALFF or fALFF values (all *p* > 0.05).

Patients with acromegaly exhibited decreased ReHo values in the left SFGdor, MFG, triangular part of the inferior frontal gyrus (IFGtriang), and left orbital part of the inferior frontal gyrus (ORBinf.L). However, ReHo values were significantly increased for the calcarine fissure and surrounding cortex (CAL) and PCUN (Table S1 and Figure 2G–I).

### 3.3 Network Topological Properties of the Functional Brain Connectome

**FIGURE 3:**
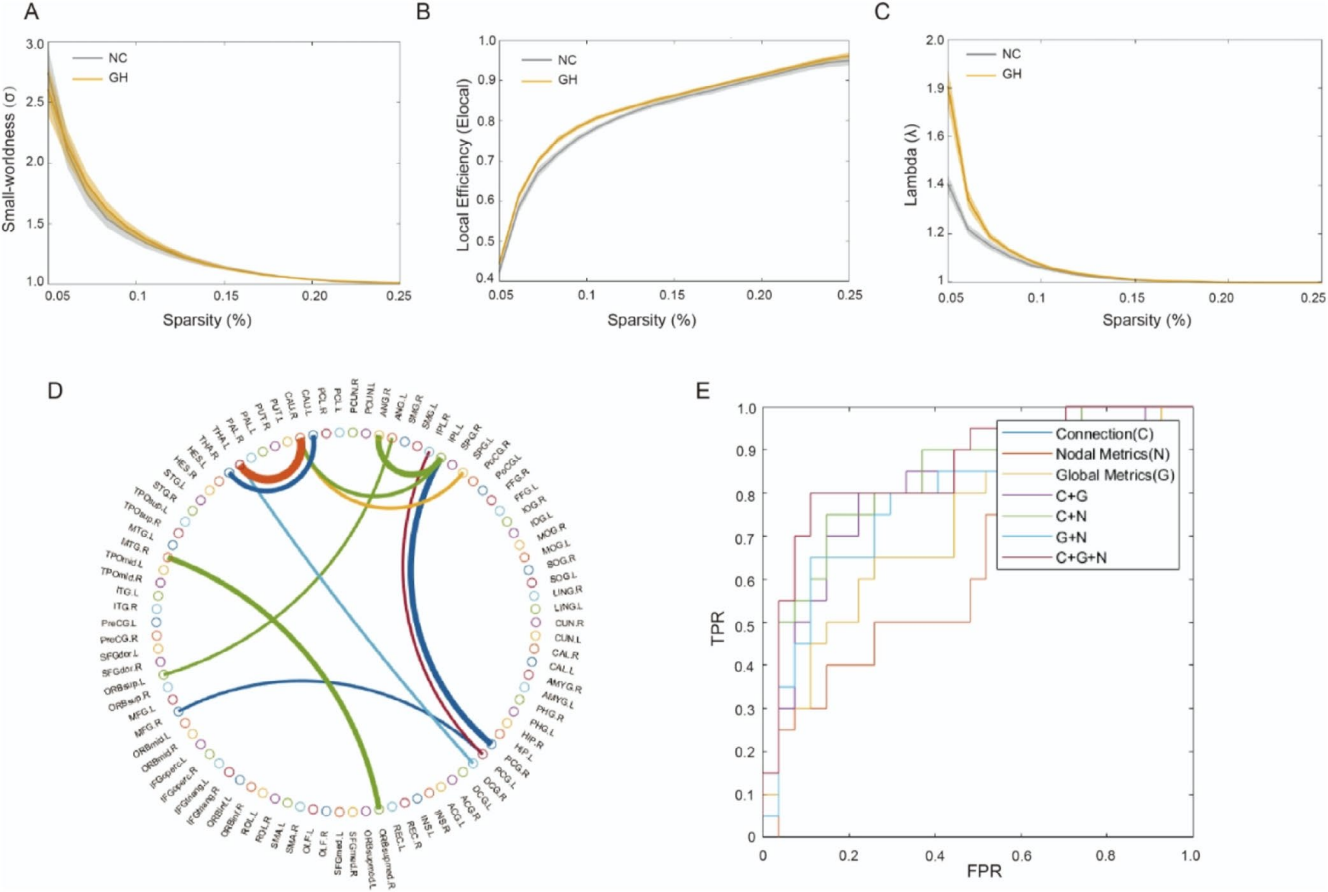
Analysis of functional brain networks. (A–C) showed the curve of small‐worldness (A), local efficiency (B), and lambda (C) of the brain function network with sparsity in acromegaly (GH) and healthy controls (HC). (D) The consensus connections of functional brain network between acromegaly and healthy controls based on AAL90. The thickness of the arcs represents the discriminative power of the connections, which is inversely related to the *p*‐values. The colors are randomly generated to differentiate individual regions of interest (ROIs). (E) Receiver operating characteristic of classification based on different features. C, connection; FPR, false‐positive rate; G, global metrics; N, nodal metrics; TPR, true‐positive rate.

Both groups exhibited small‐world organization (*σ* > 1) across the sparsity thresholds (0.05 ≤ S ≤ 0.50; Figure 3A). However, global metrics analysis revealed higher *E*_*local*_ (*p* = 0.031) and elevated *λ* (*p* = 0.004) in the acromegaly group (Table 2 and Figure 3B,C). Graph theory analysis revealed complex reorganization of nodal properties in acromegaly patients.

**TABLE 2:** Comparison of global graph metrics in the functional brain connectome between the two groups.

Global graph metrics	GH	NC	*p*
*Q*	3.84 ± 0.64	3.73 ± 0.72	0.556
*E*_global_	0.63 ± 0.02	0.62 ± 0.02	0.318
*E*_local_	0.75 ± 0.01	0.74 ± 0.02	0.031
*C*_*p*_	0.65 ± 0.03	0.64 ± 0.03	0.11
*γ*	1.29 ± 0.15	1.24 ± 0.15	0.229
*λ*	0.97 ± 0.02	0.95 ± 0.02	0.004
*σ*	1.17 ± 0.14	1.16 ± 0.14	0.779
*L*_*p*_	1.48 ± 0.09	1.46 ± 0.07	0.695

*Note:* Data are presented as the mean ± SD. Student's *t*‐test.

Abbreviations: GH, growth hormone; NC, normal controls.

Regarding centrality changes, betweenness centrality was decreased in the limbic regions (left parahippocampal gyrus, ORBsupmed) but increased in the sensorimotor nodes (right insula, left thalamus/postcentral gyrus; all *p* < 0.05). Prefrontal hubs showed reduced degree centrality in the superior parietal gyrus (*p* = 0.047) and right ORBsup (*p* = 0.019), but the nodal clustering coefficient was elevated in the left SFGdor, left angular gyrus (ANG), left ACG, and bilateral media of the superior frontal gyrus (SFGmed) (all *p* < 0.05). Regarding the clustering coefficient, the memory‐related regions (left hippocampus, *p* < 0.05) and right superior parietal gyrus (SPG) had reduced efficiency (*p* = 0.009), whereas the left postcentral gyrus (*p* = 0.038) and prefrontal regulatory nodes (bilateral SFGmed, left SFGdor, ACG) had enhanced efficiency (all *p* < 0.05; Table 3).

**TABLE 3:** Comparison of nodal graph metrics in the functional brain connectome between the two groups.

Nodal graph measures	Brain regions	Acromegaly	Controls	*p*
Betweenness	PHG.L	8.88 ± 10.47	17.30 ± 16.34	0.037
Betweenness	ORBsupmed.L	14.58 ± 10.00	22.11 ± 15.29	0.047
Betweenness	INS.R	18.03 ± 17.04	8.90 ± 6.80	0.029
Betweenness	THA.L	17.23 ± 15.13	9.05 ± 8.89	0.037
Betweenness	CUN.R	7.99 ± 6.61	15.40 ± 13.91	0.019
Betweenness	CUN.L	10.65 ± 7.84	19.74 ± 18.54	0.027
Betweenness	PoCG.L	22.14 ± 11.04	13.71 ± 11.71	0.015
Coefficient	SFGdor.L	0.30 ± 0.05	0.26 ± 0.02	< 0.001
Coefficient	PAL.R	0.27 ± 0.07	0.22 ± 0.09	0.041
Coefficient	HIP.L	0.14 ± 0.11	0.22 ± 0.10	0.013
Coefficient	ANG.L	0.29 ± 0.04	0.26 ± 0.06	0.027
Coefficient	SFGmed.L	0.29 ± 0.04	0.26 ± 0.05	0.021
Coefficient	SFGmed.R	0.32 ± 0.04	0.28 ± 0.06	0.004
Coefficient	TPOsup.R	0.27 ± 0.04	0.24 ± 0.04	0.020
Coefficient	ACG.L	0.26 ± 0.04	0.23 ± 0.04	0.008
Degree centrality	SPG.R	9.92 ± 3.72	12.22 ± 3.93	0.047
Degree centrality	ORBsupmed.R	6.35 ± 3.59	9.21 ± 4.49	0.019
Nodal efficiency	SPG.R	0.24 ± 0.03	0.26 ± 0.02	0.009
Nodal efficiency	SMG.R	0.24 ± 0.04	0.26 ± 0.03	0.046
Nodal efficiency	PoCG.L	0.29 ± 0.02	0.28 ± 0.02	0.038
Nodal local efficiency	SFGdor.L	0.37 ± 0.03	0.33 ± 0.03	0.001
Nodal local efficiency	IFGoperc.R	0.34 ± 0.05	0.36 ± 0.02	0.046
Nodal local efficiency	IPL.L	0.35 ± 0.03	0.36 ± 0.03	0.028
Nodal local efficiency	HIP.L	0.17 ± 0.13	0.28 ± 0.11	0.019
Nodal local efficiency	SFGmed.L	0.36 ± 0.03	0.33 ± 0.04	0.033
Nodal local efficiency	SFGmed.R	0.38 ± 0.03	0.35 ± 0.05	0.006
Nodal local efficiency	ACG.L	0.34 ± 0.03	0.31 ± 0.05	0.012
Nodal local efficiency	SMG.R	0.36 ± 0.04	0.38 ± 0.03	0.044

*Note:* Data are presented as the mean ± SD. Student's *t*‐test.

### 3.4 Consensus Connections of the Functional Brain Network

A total of 11 consensus connections were consistently selected across all iterations, predominantly involving the Default Mode Network (DMN), Fronto‐Parietal Control Network (FPCN), Dorsal Attention Network (DAN), and Subcortical Network (Figure 3D and Table S2).

Six connections involved the DMN or FPCN. While three intra‐DMN links (e.g., IPL.L to ANG.R; PCG.R to IPL.L) were significantly enhanced in the acromegaly group, two long‐range connections involving the orbitofrontal cortex (ORBsupmed.R and ORBsup.L) demonstrated significant hypoconnectivity. Additionally, one cross‐network control connection, linking the FPCN node (MFG.R) to the DMN node (PCG.L), exhibited increased strength.

Regarding subcortical circuitry, five connections involving the Thalamus and Caudate nucleus showed stronger positive connectivity in patients. Notably, connections between cortical nodes (IPL/SPG) and the Caudate nucleus (CAU) shifted from negative correlations in healthy controls to positive values in patients, indicating a loss of functional segregation between the cortical control systems and subcortical loops.

### 3.5 Classification

The various brain network features were used for classification. The functional connections (C), global metrics (G), and nodal metrics (N) of the brain network had AUCs of 0.811, 0.63, and 0.71, respectively. Machine learning classification using MK‐SVM (i.e., the combination of all brain network connectome features) achieved the greatest classification performance, with an accuracy of 85.11%, sensitivity of 80.00%, and an AUC of 0.86 (Figure 3E and Table S3).

## 4 Discussion

This study provides a comprehensive characterization of functional brain alterations in patients with acromegaly at rest, revealing distinct network‐level signatures of chronic GH/IGF‐1 excess. By integrating amplitude‐based (ALFF/fALFF), regional (ReHo), and topological analyses, this study revealed that GH overexposure induces multiscale functional reorganization, which includes local hyperactivity, global network fragmentation, and disrupted inter‐network integration. The machine learning model (AUC = 0.86) revealed that these multimodal features capture the neurobiological imprint of GH toxicity, offering a novel framework for understanding interactions between the endocrine system and the brain.

Patients with acromegaly often experience cognitive deterioration, but the underlying mechanisms remain unclear. Although some studies have attributed this cognitive decline to comorbidities such as sleep apnea, cerebral microbleeds, or diabetes [3, 31, 32], recent evidence has implicated the direct involvement of elevated GH and IGF‐1 levels. Elevated GH levels can affect neurotransmission and synaptic plasticity, which can lead to neuroinflammation and hippocampal dysfunction in the setting of chronic exposure [33, 34, 35]. In line with this hypothesis, patients with acromegaly had significantly decreased serum BDNF levels and increased proBDNF levels, which may indicate disrupted neurotrophic signaling and enhanced neurodegenerative processes [7, 8, 36]. Considering the established role of BDNF in learning, memory, and synaptic plasticity, this imbalance could further contribute to the observed cognitive dysfunction. Supporting these theories, the patients with acromegaly in this study had significant brain functional changes, particularly in areas related to cognitive and emotional processing. However, GH/IGF‐1 levels had no direct correlation with cognitive performance, suggesting that the effects on brain function may not be solely determined by the hormonal burden at the time of scanning. Instead, it may be the result of cumulative, long‐term neurotoxicity or irreversible structural changes incurred over the disease course. In contrast, we found significant correlations between serum BDNF, proBDNF levels and MoCA scores, suggesting that these neurotrophic signaling deficits may be linked to the cognitive decline observed in acromegaly patients. While age is a known risk factor for cognitive impairment [3], it remains unclear whether the observed cognitive dysfunction is primarily due to age‐related neurobiological changes or the long‐term effects of hormonal dysregulation. Further studies are needed to precisely clarify their relative contributions.

In recent years, neuroimaging studies have demonstrated changes in the macroscopic and microscopic brain structures in acromegaly. Some have highlighted increases in the gray and white matter, particularly in the bilateral hippocampus, which are noticeable even in the early stages of the disease [37, 38]. Due to the use of rs‐fMRI, the present study may provide new insights into changes in brain functional activity. The ALFF represents the amplitude of the BOLD signal within the low‐frequency range (typically 0.01–0.08 Hz), which reflects the intensity of spontaneous neural activity in certain brain regions at rest. Meanwhile, fALFF is a standardized form of ALFF, and changes in fALFF values mainly indicate changes in brain activity patterns rather than simply the intensity of activity [39]. The orbitofrontal cortex (ORBsup, ORBmid, and ORBinf) and ACG in patients with acromegaly had increased ALFF values but lower fALFF values, which is consistent with other research that has shown decreased prefrontal cortex activity [40]. Therefore, despite the increased intensity of spontaneous brain activity, the overall functional coordination between regions may be disrupted, particularly in areas implicated in emotion regulation and executive function.

The topological analysis of brain networks revealed that global and local network organization are altered in patients with acromegaly. Particularly, they had higher *Cp*, indicating an increase in local connectivity [41], which could reflect a compensatory mechanism for preserving network integrity. Additionally, the topological changes at the nodal level emphasize the complexity and multifaceted impact on the brain. Consistent with the results of brain functional activity, patients with acromegaly also showed a significant decrease in the efficiency and centrality of key network hubs in the DMN, visual network, and FPCN. While functional alterations in visual regions can be confounded by optic chiasm compression [42], we note that our cohort consisted of patients without radiological evidence of chiasmal compression. Recent studies reported that due to GH excess, patients demonstrated retinal nerve fiber layer thinning and microvascular changes even in the absence of compression [43]. Therefore, we hypothesize that visual network finding reflects a systemic neuroendocrine consequence of GH excess toxicity on the visual pathway, rather than a secondary degenerative effect. These changes in global and local network characteristics indicate potential compensation mechanisms and functional impairments, which can serve as the foundation for future research on the neurobiology of the disease.

The consensus connections with significant differences were mainly concentrated within the DMN and subcortical networks, as well as between the DMN and the dorsal attention network/FPCN. This discovery provides further insight into the neural mechanisms underlying cognitive dysfunction in acromegaly. The DMN is highly active in the resting state and is involved in higher cognitive functions such as self‐referential thinking, episodic memory retrieval, and future planning [44, 45]. Abnormal connectivity within this network can result in symptoms commonly seen in patients, such as memory impairment, slowed thinking, and social cognitive deficits [46]. On the other hand, the subcortical network, which includes structures such as the basal ganglia and thalamus, plays a central role in motor control, emotional regulation, and hormonal feedback loops (e.g., the HPA axis) [47, 48]. Changes in its connectivity can affect motor coordination and emotional states, and these may be either directly or indirectly related to the core pathology of acromegaly, which involves the chronic neurotoxic effects of long‐term GH and IGF‐1 excess on subcortical structures [49, 50]. More importantly, there is typically an inverse correlation between the DMN and the FPCN/dorsal attention network; this balance is crucial for the flexible allocation of cognitive resources [51, 52]. In this study, significant alterations were found in the connectivity both within and between these key networks. This strongly suggests that the cognitive deficits in patients with acromegaly are caused by the decreased efficiency of information integration within multiple large‐scale brain networks and disrupted dynamic interactions between these networks. These widespread abnormalities in network topology may be the cumulative result of the chronic effects of high GH/IGF‐1 levels on neurons, glial cells, and cerebral vasculature, ultimately impairing the overall efficacy and resilience of neural network functioning [4]. This information lays a valuable theoretical foundation for future studies on the neurotoxic targets of excess GH/IGF‐1, the development of targeted neuroprotective or cognitive rehabilitation strategies, and the utilization of these network metrics as biomarkers for disease progression or treatment efficacy.

Given the relatively small sample size and large number of derived features, we employed a nested LOOCV strategy, in which feature selection and parameter tuning were performed strictly within the training set to prevent data leakage and minimize potential overfitting. The identification of Consensus Connections further supports the stability of this underlying network signature. Although our model achieved an accuracy of 85.11% in distinguishing patients from controls, this result should be interpreted cautiously. The current performance mainly demonstrates the potential discriminative information contained in the functional connectivity features rather than a clinically applicable diagnostic tool. Emerging evidence also suggests that neuroimaging, particularly fMRI, can provide valuable insights into the pathophysiology of endocrine disorders and may be useful for monitoring disease impact on brain function.

Despite these compelling findings, this study has several limitations. Given the limited sample size and the exploratory nature of the study, we chose a relatively lenient statistical threshold to detect the subtle yet meaningful neural alterations. The lack of external validation limits the generalizability of the model. These results need further validation in larger, multicenter cohorts using more stringent correction methods (voxel‐level *p* < 0.001 and cluster‐level *p* < 0.05). Beyond static analysis in the study, dynamic functional connectivity mapping can be complementary to offer a comprehensive mechanistic framework for understanding functional network deficits [53, 54, 55]. Moreover, it remains unclear whether these observed changes in brain function are reversible with treatment or progress over time, thereby warranting further investigation with longitudinal studies assessing the effects of treatment on brain activity and cognitive function.

## 5 Conclusion

In conclusion, this study provides evidence of significant functional brain changes in acromegaly, particularly in networks related to cognitive and emotional processing. These results offer novel insight into the mechanisms of excess GH and IGF‐1 in the brain.

## Author Contributions

W.H. and Z.M. conceived the study and supervised the overall research design. Q.Z., S.Y., X.S., X.Z., Y.W., H.L., L.J., Y.Y., and W.W. contributed to participant recruitment, clinical evaluation, and acquisition of imaging and clinical datasets. Z.W., X.Y., and J.Y. performed the initial fMRI preprocessing and statistical analysis. Y.Z. additionally conducted major re‐analyses, optimization of analytical pipelines, and methodological refinement during the revision stage. Z.W. drafted the original manuscript. Y.Z. and X.Y. contributed substantially to the interpretation of results and major revision of the manuscript. All authors approved the final manuscript.

## Funding

This study was supported by the National Natural Science Foundation of China (Grant 82371875); Zhejiang Provincial Natural Science Foundation of China (Grant LY22H160016).

## Disclosure

The authors have nothing to report.

## Ethics Statement

This study was approved by the Ethics Committee of Huashan Hospital, Fudan University (KY2015‐256) in accordance with the Declaration of Helsinki. All participants provided written informed consent.

## Conflicts of Interest

The authors declare no conflicts of interest.

## Supporting information


**Table S1:** Acromegaly‐related alterations in ALFF, fALFF and ReHo.

**Table S2:** Consensus connections of the functional brain network.

**Table S3:** Classification performance corresponding to different functional connectome features.

## Data Availability

All datasets generated during and/or analyzed during the current study are not publicly available but are available from the corresponding author on reasonable request.
