# Supplementary material for: Altered Brain Function and Network Topology in Patients With Acromegaly: Resting‐State fMRI Study of Networks Related to Cognitive and Emotional Processing
Source: CNS Neurosci Ther. 2026 Jan 19;32(1):e70755. doi: 10.1002/cns.70755 (PMC12813690; doi:10.1002/cns.70755)
Supplement: Supplementary file 1 — Table S1: Acromegaly‐related alterations in ALFF, fALFF and ReHo. [file CNS-32-e70755-s003.docx]

**Table S1. Acromegaly-related alterations in ALFF, fALFF and ReHo**

| Item | Cluster | Peak MNI coordinate | | | T-value | Main brain regions included (AAL) | Number of voxels |
| --- | --- | --- | --- | --- | --- | --- | --- |
|  |  | x | y | z |  |  |  |
| ALFF | Cluster1 | 6 | 33 | −9 | 4.5112 | Frontal_Med_Orb_R | 97 |
|  |  |  |  |  |  | Rectus_R | 93 |
|  |  |  |  |  |  | Cingulum_Ant_R | 87 |
|  |  |  |  |  |  | Frontal_Sup_Orb_R | 78 |
|  |  |  |  |  |  | Frontal_Inf_Orb_R | 64 |
|  |  |  |  |  |  | Frontal_Mid_Orb_R | 46 |
| fALFF | Cluster2 | 12 | 21 | -12 | −5.1979 | Frontal_Mid_L | 397 |
|  |  |  |  |  |  | Frontal_Sup_L | 324 |
|  |  |  |  |  |  | Frontal_Mid_R | 288 |
|  |  |  |  |  |  | Frontal_Sup_R | 204 |
|  |  |  |  |  |  | Frontal_Mid_Orb_R | 120 |
|  |  |  |  |  |  | Frontal_Sup_Medial_L | 77 |
|  |  |  |  |  |  | Cingulum_Ant_L | 65 |
|  |  |  |  |  |  | Frontal_Sup_Medial_R | 61 |
|  |  |  |  |  |  | Frontal_Mid_Orb_L | 59 |
|  |  |  |  |  |  | Frontal_Inf_Orb_L | 56 |
|  |  |  |  |  |  | Frontal_Sup_Orb_R | 52 |
|  |  |  |  |  |  | Rectus_L | 52 |
|  |  |  |  |  |  | Frontal_Med_Orb_L | 48 |
|  |  |  |  |  |  | Frontal_Sup_Orb_L | 48 |
|  |  |  |  |  |  | Frontal_Inf_Tri_L | 36 |
|  |  |  |  |  |  | Rectus_R | 33 |
|  | Cluster3 | -12 | −39 | 27 | −3.4297 | Precuneus_R | 116 |
| ReHo | Cluster4 | −27 | 36 | 0 | −3.4288 | Frontal_Sup_L | 104 |
|  |  |  |  |  |  | Frontal_Inf_Tri_L | 93 |
|  |  |  |  |  |  | Frontal_Mid_L | 88 |
|  |  |  |  |  |  | Frontal_Inf_Orb_L | 68 |
|  | Cluster5 | −24 | −63 | 0 | 4.81 | Calcarine_L | 152 |
|  |  |  |  |  |  | Precuneus_L | 105 |
|  |  |  |  |  |  | Occipital_Sup_L | 101 |
|  |  |  |  |  |  | Occipital_Mid_L | 100 |
|  |  |  |  |  |  | Cuneus_L | 85 |
|  |  |  |  |  |  | Cuneus_R | 83 |
|  |  |  |  |  |  | Lingual_L | 65 |
|  |  |  |  |  |  | Cingulum_Mid_L | 44 |
|  |  |  |  |  |  | Occipital_Sup_R | 42 |

Abbreviations: ALFF, amplitude of low-frequency fluctuations; fALFF, fractional ALFF; ReHo, regional homogeneity; AAL, anatomical automatic labeling; MNI: Montreal Neurological Institute.
