# Supplementary material for: Altered Brain Function and Network Topology in Patients With Acromegaly: Resting‐State fMRI Study of Networks Related to Cognitive and Emotional Processing
Source: CNS Neurosci Ther. 2026 Jan 19;32(1):e70755. doi: 10.1002/cns.70755 (PMC12813690; doi:10.1002/cns.70755)
Supplement: Supplementary file 2 — Table S2: Consensus connections of the functional brain network. [file CNS-32-e70755-s001.docx]

**Table S2. Consensus connections of the functional brain network**

| **Connection  (Node A-Node B)** | **Network** | **P-value** | **Mean FC  (HC)** | **Mean FC  (Acro)** | **Alteration** |
| --- | --- | --- | --- | --- | --- |
| **Subcortical Network** | | | | | |
| CAU.R – THA.L | SUB – SUB | 0.0018 | 0.24 | 0.42 | Hyperconnectivity |
| CAU.L – THA.R | SUB – SUB | 0.0032 | 0.19 | 0.37 | Hyperconnectivity |
| DCG.R – THA.L | VAN – SUB | 0.0058 | 0.26 | 0.43 | Hyperconnectivity |
| IPL.L – CAU.R | DMN – SUB | 0.0044 | -0.14 | 0.08 | Hyperconnectivity |
| SPG.L – CAU.R | DAN – SUB | 0.0049 | -0.13 | 0.07 | Hyperconnectivity |
| **Default Mode Network** | | | | | |
| IPL.L – ANG.R | DMN – DMN | 0.0025 | 0.35 | 0.54 | Hyperconnectivity |
| PCG.R – IPL.L | DMN – DMN | 0.0025 | 0.14 | 0.34 | Hyperconnectivity |
| PCG.L – IPL.R | DMN – DMN | 0.0066 | 0.12 | 0.36 | Hyperconnectivity |
| ORBsupmed.R – MTG.R | LIM/DMN – DMN | 0.003 | 0.53 | 0.38 | Hypoconnectivity |
| ORBsup.L-ANG.L | LIM/FPCN – DMN | 0.0056 | 0.5 | 0.32 | Hypoconnectivity |
| **Network Interaction** | | | | | |
| MFG.R – PCG.L | FPCN – DMN | 0.0057 | 0.24 | 0.42 | Hyperconnectivity |

FC, Functional Connectivity; HC, Healthy Controls; Acro, Acromegaly Patients; CAU, Caudate; THA, Thalamus; DCG, Mid Cingulate Gyrus; IPL, Inferior Parietal Lobule; SPG, Superior Parietal Gyrus; ANG, Angular Gyrus; PCG, Posterior Cingulate Gyrus; ORBsupmed, Superior Frontal Gyrus, medial orbital part; MTG, Middle Temporal Gyrus; MFG, Middle Frontal Gyrus. Networks: SUB, Subcortical; DMN, Default Mode Network; FPCN, Fronto-Parietal Control Network; DAN, Dorsal Attention Network; VAN/SAL, Ventral Attention/Salience Network; LIM, Limbic Network.
