# Supplementary material for: Altered Brain Function and Network Topology in Patients With Acromegaly: Resting‐State fMRI Study of Networks Related to Cognitive and Emotional Processing
Source: CNS Neurosci Ther. 2026 Jan 19;32(1):e70755. doi: 10.1002/cns.70755 (PMC12813690; doi:10.1002/cns.70755)
Supplement: Supplementary file 3 — Table S3: Classification performance corresponding to different functional connectome features. [file CNS-32-e70755-s002.docx]

**Table S3. Classification performance corresponding to different functional connectome features**

| **Method** | **Accuracy (%)** | **Sensitivity (%)** | **Specificity (%)** | **AUC** |
| --- | --- | --- | --- | --- |
| Connection (C) | 74.47 | 70.00 | 77.78 | 0.811 |
| Nodal (N) | 65.96 | 40.00 | 85.19 | 0.626 |
| Global (G) | 65.96 | 50.00 | 77.78 | 0.713 |
| C+N | 82.98 | 80.00 | 85.19 | 0.831 |
| C+G | 80.86 | 75.00 | 85.19 | 0.811 |
| N+G | 76.60 | 60.00 | 88.89 | 0.796 |
| C+N+G | 85.11 | 80.00 | 88.89 | **0.856** |
